# A national cross-sectional analysis of stakeholder views regarding the practice and governance of robotic surgery

**DOI:** 10.1007/s11701-025-02354-w

**Published:** 2025-05-06

**Authors:** Stefanie M. Croghan, Christina A. Fleming, Ruaidhrí McVey, Tom Moran, Gary Fitzmaurice, Emeka Okereke, Dara A. O.’ Keeffe, Fiachra E. Rowan, Kevin Barry, Barry B. McGuire, RCSI National Leads in Robotic Surgery

**Affiliations:** 1 Department of Surgical Affairs, Royal College of Surgeons in Ireland, Dublin, Ireland; 2 Department of Urology and Transplantation, Beaumont Hospital, Dublin, Ireland; 3 Department of Surgery, University Hospital Limerick, Limerick, Ireland; 4 School of Medicine, University of Limerick, Limerick, Ireland; 5 Department of Gynaecology, St. Vincent’s University Hospital, Dublin, Ireland; 6 Department of Gynaecology, Mater Misericordiae University Hospital, Dublin, Ireland; 7 Department of Otolaryngology and Head & Neck Surgery, St. Vincent’s University Hospital, Dublin, Ireland; 8 Department of Otolaryngology and Head & Neck Surgery, Mater Misericordiae University Hospital, Dublin, Ireland; 9 Department of Cardiothoracic Surgery, St. James’s Hospital, Dublin, Ireland; 10 Trinity St. James’s Cancer Institute, Dublin, Ireland; 11 Department of Orthopaedic Surgery, University Hospital Waterford, Waterford, Ireland; 12 Department of Surgery, Mayo University Hospital, Castlebar, Mayo, Ireland; 13 Department of Urology, St. Vincent’s University Hospital, Dublin, Ireland

**Keywords:** Robotic surgery, Stakeholders, Governance, Case volume, Indicative, Index procedures, Key performance indicators (KPIs), Safety, Ireland, Training, Future planning, Communication, Team work

## Abstract

The study objective was to explore the views of key national stakeholders regarding the practice and governance of robotic surgery in Ireland. A literature review and analysis by two expert robotic surgeons was used to design a customised questionnaire, interrogating respondent perceptions of key robotic surgical areas, including surgical practice, governance, audit and key performance indicators (KPIs). The questionnaire was disseminated via specialty representatives, inviting responses from robotic surgeons, senior trainees, bedside assistants, clinical nurse managers, hospital management and industry partners. The responses were compiled and analysed, with thematic analysis applied to free-text data. Responses were received from 87 stakeholders, with representation from all invited professional groups, surgical specialties and national geographical regions. The largest respondent cohort was consultant robotic surgeons (*n* = 61); estimated response rate 76.3%. Most respondents were in favour of proctorship/mentorship arrangements for early-career surgeons, with support expressed for external proctors (80.5%, *n* = 70), internal proctors (88.5%, *n* = 77) and peer-to-peer mentoring (87.4%, *n* = 76). The minimum average number of robotic cases necessary for a trained surgeon to maintain competency was most frequently perceived as 1 per fortnight (39.3%, *n* = 24), or 1 per week (37.7%, *n* = 23). The recording of KPIs was believed to be important at both unit level [93.1% (*n* = 81) agree] and surgeon level (94.3% (*n* = 82) agree). Ninety-five percent of respondents (*n* = 83), thought institutions should have robotic governance committees. Strong support [83.9% (*n* = 73)] was expressed for the creation of a national guideline or governance document for the practice of robotic surgery. This national, pan-specialty robotic surgery stakeholder survey is the first of its kind. It identifies strong national support for continued advances in the training and governance of robotic surgery, with consensus for institutional oversight of robotic surgery and the development of a national guidelines for the conduct of robotic surgery.

**Supplementary Information:**

The online version contains supplementary material available at 10.1007/s11701-025-02354-w.

## Introduction

Robotic surgery has seen rapid expansion in recent years. This is evidenced by its 2024 global market value of $9.1 billion, with a predicted compound annual growth rate (CAGR) > 8% [1] and an estimated > 900 new robotic platforms becoming operational each year worldwide [2]. Robotic surgery, as compared to traditional open approaches, offers numerous potential advantages to patients, surgeons, hospital systems, and societies. These include reduced perioperative blood loss, decreased postoperative pain scores, shortened length of hospital stay and faster return to work [3]. In addition, a greater magnification and enhanced visualisation of the surgical site, tremor elimination and increased ergonomic efficiency may improve precision and help to reduce surgical error [3]. Accordingly, surgical robots have been introduced to most surgical subspecialties, with widespread uptake in many of these [4].

However, any new technology, particularly one that is so rapidly and radically changing the surgical landscape, requires careful analysis and close oversight to ensure its safe and effective integration into clinical practice. The robotic surgical systems represent highly sophisticated and complex technological innovations. Accordingly, their safe and effective use demands comprehensive specialised training for a range of staff members. As well as being capable of robotically performing, or assisting with, specific surgical procedures, the surgical team must become proficient in operating the robotic system itself, capable of troubleshooting errors and acquainted with a new range of instruments. It has been demonstrated that laparoscopic surgical skills are not directly transferrable to robotic platforms, confirming that dedicated robotic console training is required even for surgeons competent in other surgical approaches to a particular procedure [5]. Notably, although perhaps not surprisingly, several studies have suggested an increased risk of perioperative complications during the introduction phase of novel technology to operating theatre environments [6–8].

It is clear that adequate governance is required to optimise the many potential benefits stemming from the introduction of robotic surgery, whilst ensuring patient safety at all stages of the introduction and maintenance of an institution’s robotic surgical programme [8]. At present, in the Republic of Ireland, there has been widespread adoption, with plans for further expansion. However, governance and credentialing are currently at the discretion of individual institutions and industry partners, in the absence of a national framework.

Early input from relevant parties is critical to guide the development, implementation and monitoring of any new innovation in healthcare, with stakeholder engagement an integral part of guideline development [9]. In this study, we aimed to explore the perspectives of national stakeholders on the current state and oversight of robotic surgery in Ireland, with a focus on governance, training and future direction.

## Methodology

### Overview

A customised online questionnaire was created, exploring respondent perceptions of key areas relevant to robotic surgery and their attitudes towards appropriate training, utilisation and governance structures. This questionnaire was used to conduct a cross-sectional analysis of members of the robotic surgical team and disseminated nationally in Ireland. The questionnaire was designed to collect both quantitative and qualitative data, with free-text boxes incorporated.

### Hypothesis and outcome measures

We hypothesised that robotic stakeholders in Ireland would be in favour of formal governance and training structures pertaining to robotic surgery. The key outcome measures included:

- The minimum annual case volume that robotic surgeons considered necessary to maintain proficiency
- The level of support and/or disagreement expressed by robotic stakeholders for proctorship and mentoring arrangements
- The level of support and/or disagreement expressed by robotic stakeholders for the recording of key performance indicators (KPIs) at unit level, and at surgeon level
- The level of support and/or disagreement expressed by robotic stakeholders on institutional robotic governance committees and a national guideline document pertaining to robotic surgery.

### Questionnaire design and pilot

A multi-step process was used to design the questionnaire used. Both a background literature review and informal interviews with robotic surgeons, surgical trainees, clinical nurse managers and bedside assistants were performed (SC, CF) to inform questionnaire content. Thereafter, a survey questionnaire was drafted (SC) and further refined in an iterative process (CF, BM). The resultant questionnaire, hosted online by Google™ Forms, underwent pilot testing with five members of the robotic surgical team, to ensure comprehensibility and feasibility of completion.

The finalised questionnaire (Supplementary file 2: Appendix 1) contained 31 items spanning domains of: respondent demographics, surgical practice, case volume, proctorship and mentoring, operational governance, clinical governance, audit and key performance indicators.

### Sampling approach and inclusion criteria

An electronic link to the questionnaire was created. This was shared via the National Leads on Robotic Surgery Group at the Royal College of Surgeons in Ireland (RCSI). The representative leads for each surgical subspecialty ensured dissemination of the questionnaire amongst robotic surgeons and trainee members of their specialty, and a representative for each public hospital shared the link amongst robotic nursing staff, robotic bedside assistants, members of hospital management and industry representatives. All members of the aforementioned groups, who deemed themselves to have involvement with robotic surgery, were eligible to participate. For the purpose of this study, ‘robotic surgeons’ are considered to be surgeons who have completed their specialist training and are working at ‘consultant’ (‘attending’) level, with or without ongoing mentorship/proctorship arrangements.

### Data collection

The survey link directed potential participants to a web page containing further study information, eligibility criteria, and investigator contact details, and allowing the viewer to proceed to the survey questions (Supplementary file 2: Appendix 1). Consent to data processing of a respondent’s response was implied by his/her completion of the questionnaire. The survey responses were collated and stored in a confidential manner, with access available to the investigators only.

### Data analysis

Data were downloaded and aggregate data compiled in Microsoft® Excel®. Descriptive statistics were used to analyse and report on demographic details and categorical responses. Subgroup analyses of associations were performed using chi-square tests. Thematic analysis, as described by Braun and Clark [10], was applied to the qualitative data generated by free-text responses. This is a recognised method for identifying and reporting upon themes that emerge from qualitative data. In this analysis, a six-step framework was applied, involving researcher familiarisation with the data, generation of initial codes, development of themes, refinement of themes, naming of themes, and writing up the qualitative findings.

## Results

### Participant characteristics

The responses were received from a total of 87 participants. The largest group of respondents was robotic surgeons (70.1%, *n* = 61), with an estimated response rate of 76.3%. Other respondent cohorts were: senior surgical trainees (13.8%, *n* = 12), robotic clinical nurse managers (5.7%, *n* = 5), hospital managerial team members (5.7%, *n* = 5), robotic bedside assistants (4.6%, *n* = 4) and industry partners (3.4%, *n* = 3). Where participants had a managerial role (e.g. clinical director) along with a personal robotic surgical practice, they were included in both cohorts in this breakdown. The personal practice figures of respondents are presented in Table 1. Urological (38.6%, *n* = 28), colorectal (26%, *n* = 19) and general surgical (16.4%, *n* = 12) robotic practices were most common amongst participants, with a wide distribution of years of experience. Approximately half of the robotic surgeons had completed a robotic surgical fellowship; this was directly correlated with years of surgical practice. Of surgeons in their first 10 years of independent practice, a robotic fellowship had been completed by 73.5% (25/34), as compared to 22.2% (6/27) of surgeons with more than 10 years experience (*p* < 0.001).

**Table 1:** Surgical experience and practice

Surgical specialty (Consultants and Trainees, *n* = 73)	Specialty	% (*n*)	
	Urology	38.6% (28)	
	Colorectal surgery	26% (19)	
	General surgery	16.4% (12)	
	Gynaecology	13.7% (10)	
	Upper GI	9.6% (7)	
	Thoracic surgery	5.5% (4)	
	Trauma/Orthopaedics	5.5% (4)	
	HPB	4.1% (3)	
	Cardiac	1.4% (1)	
	Transplant	1.4% (1)	
	ENT/Head & Neck	1.4% (1)	
	Endocrine Surgery	1.4% (1)	
Current consultants (*n* = 61)			
Years of experience	Years	As Consultant	As Robotic Consultant
		*n* (%)	*n* (%)
	0–5	20 (32.8%)	34 (55.7%)
	6–10	14 (23%)	20 (32.8%)
	11–15	10 (16.4%)	6 (9.8%)
	16–20	8 (13.1%)	1 (1.6%)
	> 20	9 (14.8%)	0
Personal case volume per year	Case Number	*n* (%)	
	< 20	8 (13.1%)	
	21–40	12 (19.7%)	
	41–50	14 (23%)	
	51–60	5 (8.2%)	
	61–70	4 (6.6%)	
	> 70	16 (%)	
	N/A (not started)	2 (26.3%)	
Formal robotic fellowship	Yes	31 (50.8%)	
	No	30 (49.2%)	
Console training received on training scheme pre-consultancy	Yes	14 (23%)	
	No	47 (77%)	
Senior Trainees (*n* = 12)			
Console training received on scheme	Yes	10 (83.3%)	
	No	2 (16.7%)	
Case Volume per Year (cases involved in)	Case Number	*n* (%)	
	< 20	4 (33.3%)	
	21–40	1 (8.3%)	
	41–50	2 (16.7%)	
	51–60	3 (25%)	
	61–70	–	
	> 70	2 (16.7%)	

It presents the characteristics and personal practice settings of the study participants

Where respondents were aware of the overall robotic surgery case volume of their institution (*n* = 43), this was typically > 100 cases per annum (reported by 88.4%, *n* = 38), with > 250 cases per annum reported by 45.2% (*n* = 20).

### Proctorship and mentorship

The study participants reported their level of agreement with the use of proctoring and mentoring systems for surgeons in their first number of robotic cases. The proctors were defined as experienced robotic surgeons attending to supervise a robotic operation. An ‘external proctor’ was defined as a surgeon coming from an outside institution and an ‘internal proctor’ was defined as a surgeon practicing in the same department as the surgeon being proctored. Peer-to-peer mentoring was defined as a more formal relationship, where a colleague within the same institution mentors the surgeon early-on in their learning curve until he/she is signed off as proficient. The group consensus was largely in favour of the outlined proctorship and mentorship arrangements (Table 2), with the majority expressing positive agreement (‘agree’ or ‘strongly agree’) with the use of external proctors (80.5%, *n* = 70), internal proctors (88.5%, *n* = 77) and peer-to-peer mentors (87.4%, *n* = 76).

**Table 2:** Attitudes towards proctorship and mentorship in robotic surgery

Concept	Strongly Agree % (*n*)	Agree % (*n*)	Neutral % (*n*)	Disagree % (*n*)	Strongly Disagree % (*n*)	Unsure % (*n*)
External proctor	43.7% (38)	36.8% (32)	10.3% (9)	3.4% (3)	3.4% (3)	2.3% (2)
Internal proctor	36.8% (32)	51.7% (45)	6.9% (6)	1.1% (1)	2.3% (2)	1% (1)
Peer-to-peer mentoring	36.8% (32)	50.6% (44)	9.2% (8)	0	1.1% (1)	2.3% (2)

It presents the level of agreement, as reported on a five-point Likert scale, of study respondents (*n* = 87), with the concepts of internal proctors, external proctors and peer-to-peer mentors as a means to supervise and/or support early-career robotic surgeons

The free-text comments raised a number of further themes related to proctoring and mentoring arrangements. The importance of proctor selection was highlighted, with one respondent expressing the option that the “ideal proctor” should be “selected by [the] surgeon, not by industry,” and emphasising the need to “ensure [the selected] proctor has experience in comparable technique/approach e.g. type of anastomosis,” as this “varies widely.” External proctors were felt to be preferable to internal proctors by some respondents, as this was seen to potentially facilitate more “objectivity,” and “maybe a more comfortable coaching/mentoring relationship in some cases.” The extent of a surgeon’s prior training was perceived as relevant in assessing the need for a proctor or mentor, with one respondent feeling that a surgeon does not need a proctor “if coming back from fellowship,” whilst acknowledging that “peer support [is] always helpful.”

### Necessary case volume

**Fig. 1:**
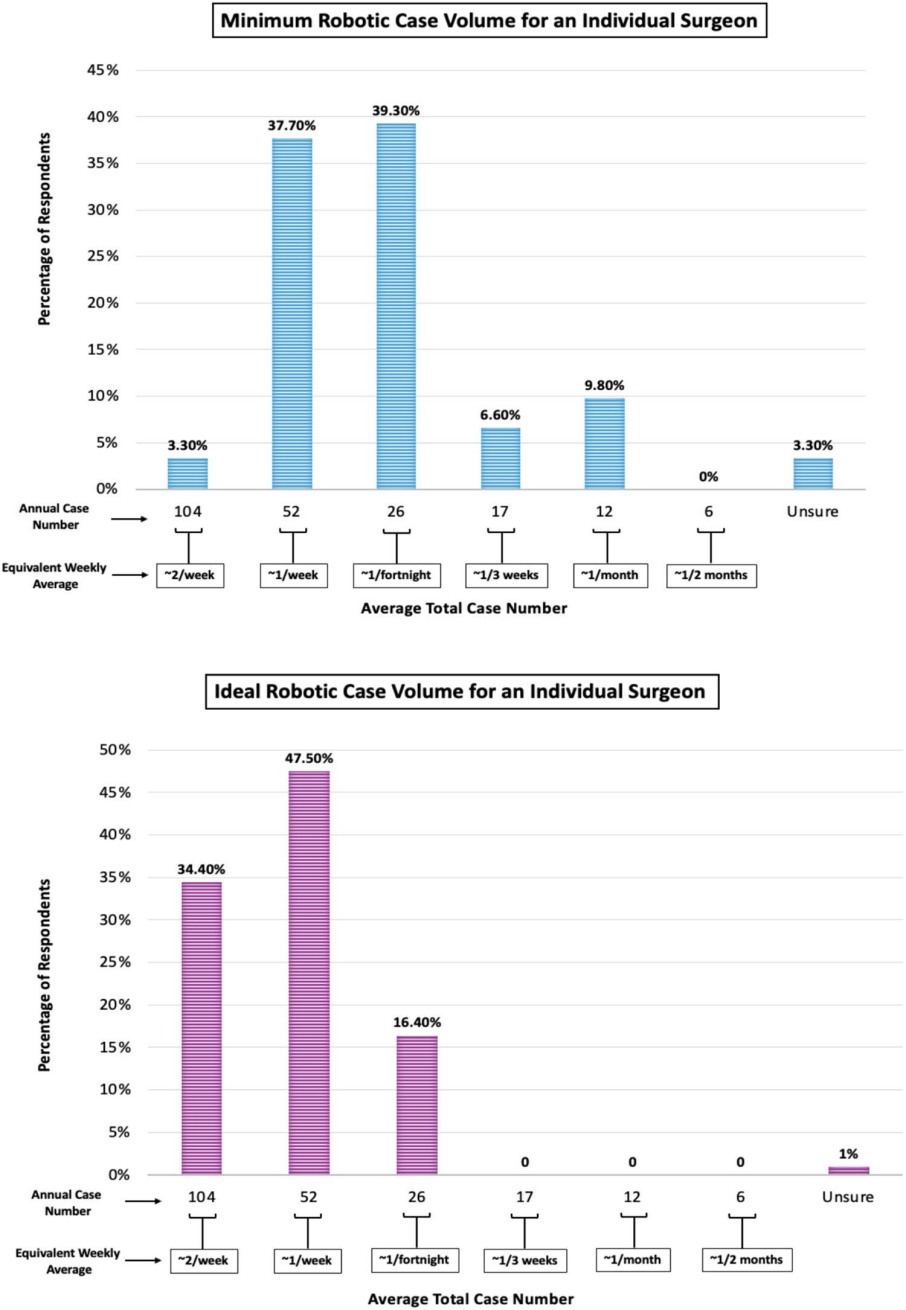
Robotic case volume. It presents the minimum and ideal robotic case volumes for an individual surgeon as perceived by surgeon respondents (*n* = 61). The percentage of respondents citing a given case volume are displayed on the y axis, while the x axis displays categories of average annual case volumes per year, with average weekly/monthly equivalents

Trained robotic surgeons (*n* = 61) were asked what they perceive to be both the minimum necessary, and the ideal, overall robotic case volume for an individual surgeon to maintain proficiency. The responses are presented in Fig. 1. The most frequently chosen minimum necessary robotic case volume was an average of 26 cases a year (approximately one every 2 weeks); as selected by 39.3% of respondents. Only 16.4% of respondents chose a lower figure as the minimum necessary case volume. With regard to the ideal average robotic case volume, almost half (47.5%) perceived this to be 52 cases per year (1 case per week), with another 34.4% reporting an average of 104 robotic cases per year (2 cases per week) to be ideal.

The qualitative data raised some challenges with defining minimum and optimal case volumes across the board. One respondent identified the “arguably…greater experience” that a surgeon may obtain from robotically completing “a major resectional case” that “will take all day,” as compared to doing “4 anti-reflux cases in the same timeframe,” thereby highlighting that “optimal case volumes” may vary according to “specialty and scope of practice.” It was also expressed that where a robotic procedure “is very similar” to its laparoscopic equivalent, a lower robotic case volume may be adequate, but that certain complex procedures require “much greater familiarity with the robotic system,” presumably needing a higher case volume.

The perception that necessary case volume may change over a surgeon’s career was also voiced, with the opinion that “small gaps between cases isn’t as big an issue if [a surgeon] has already got over the learning curve on fellowship.”

### Communication

Quite divergent responses were received to the question of whether communication to team members can be more challenging during robotic surgery than other forms of surgery. Half of all (*n* = 87) participants (50.6%, *n* = 44) disagreed that communication challenges are increased with robotic platforms, whilst 39.1% (*n* = 34) agreed that this may be the case, and 10.3% (*n* = 9) selected neutral response. On subgroup analysis of robotic surgeons (*n* = 61), no significant difference in response to this topic was seen between those with > 10 years and < 10 years of experience (*p* = 0.67).

Nonetheless, an almost unanimous feeling was expressed that specific training should be delivered to all members of the operating theatre team regarding effective communication during robotic surgery: 93.1% (*n* = 81) agreed and 6.9% (*n* = 6) were neutral, with no respondents expressing disagreement.

Some elaboration on aspects of communication was supplied in free-text data, which may explain the divergent responses above. The differences in theatre communication with robotic versus other surgical approaches were stated, with “aspects of communication [being] different on the robot—some worse, some better,” meaning that “communication [is not] necessarily worse on the robot, but needs to be trained for differently.” The opportunity for “courses to focus on how to [best] leverage” communication during robotic surgery was identified. The importance of communication, seen as “key,” was reiterated.

### Safety and emergencies

A minority of respondents (20.7%, *n* = 18) feel that robotic surgery has increased risk compared to other surgery in the event of a sudden emergency, such as unexpected major bleeding or a cardiac arrest. Most (55.2%, *n* = 48) disagreed with this viewpoint, whilst a significant portion of respondents (24.1%, *n* = 18) expressed uncertainty.

The vast majority (88.5%, *n* = 77) did express agreement that emergency undocking should be rehearsed regularly to ensure that there is an efficient robotic team response, should an emergency occur.

Additional safety initiatives were proposed from free-text responses. A suggestion was made for a “robotic surgery specific checklist to be developed, to highlight the important steps that all staff in the OR [operating room] need to be aware of, e.g. US [ultrasound] probe ready for partial nephrectomy.” In addition, the potential for utilisation “of video review” as “an important safety and learning option,” was emphasised.

### Robot system utilisation

The questionnaire explored attitudes towards utilisation of a robotic surgery operating theatre. The concept of assigning a dedicated robotic surgery operating theatre, as opposed to moving the surgical robot as required, was strongly favoured, with 89.7% (*n* = 78) in agreement.

Additional relevant factors were raised in free-text comments, including logistics and future evolutions. Whilst one respondent felt that a dedicated robotic theatre “is a must,” others expressed the view that this would “ideally be the case,” but accepted that this “may not be practical” in the “Irish context” with “other logistical pressures.” Another stressed the primary importance of ensuring that “all OR [operating room] staff involved have training on a [robotic] system, especially if moving between ORs.” Potential future evolutions were referenced, with comments that “with [new] robotic systems, moving the robot may be more practical,” and that “the future is robotic platforms in multiple theatres…we should move away from thinking about ‘the’ robot and move towards the robots.”

To optimise utilisation, most (94.3%, *n* = 82) respondents agreed that a surgical robot should be used to maximum capacity on working days.

Caveats to overly simplistic attitudes towards utilisation metrics of robotic surgical systems were, however, voiced. The concepts of staffing and surgeon capability was raised with the view that “max [robotic] capacity” can be aimed for, “as long as there are trained, competent surgeons” to deliver this. Appropriateness of case selection was also stressed, as “it would not be reasonable to try to ‘fill up’ the robotic list with cases that might be better done another way (e.g. [laparoscopic] cholecystectomy) for the purpose of ‘optimising utilisation’ alone.”

The need to prioritise safety and practicality with utilisation initiatives such as “evening operating” was also stressed. One respondent gave the example of “major resectional cases being the focus of [our upper gastrointestinal] robotic programme,” which would not be suitable as evening cases due to the inherent “long operative duration and need for specialised anaesthetic support.”

The ability to optimise utilisation by carefully considered scheduling was also raised, with the example that it may be “better for a team to do alternating robotic/regular lists” rather than “weekly half day robotic lists,” so as to “free up the robot for other specialties.”

### Audit and key performance indicators

The study respondents were largely in favour of the recording of KPIs at both unit-level and surgeon-level. Eighty-one respondents (93.1%) agreed/strongly agreed that unit-level KPIs should be recorded; 3.5% (*n* = 3) disagreed with this proposition. A strong majority (94.3%, *n* = 82) also agreed/strongly agreed with the recording of surgeon-level KPIs, while 1.1% (*n* = 1) disagreed. The opinions on which specific KPIs should be considered are presented in Table 3.

**Table 3:** Stakeholder views on KPIs

*Potential KPI * *(Proposed by Researchers)*	*Respondents Supporting Inclusion* *% (n)*
Unexpected return to the operating theatre	89.7% (78)
30-day perioperative mortality	87.4% (76)
Unexpected ICU (Intensive Care Unit) admission	79.3% (69)
30-day perioperative morbidity	78.2% (68)
Intraoperative transfusion requirement	78.2% (68)
Any surgery >6 hours duration (console time)	72.4% (63)
Conversion to open surgical approach	72.4% (63)
Conversion to laparoscopic surgical approach	49.4% (43)
*Additional KPIs Proposed (Generic)* *suggested by >1 respondent	
Infection rate *	
Docking time *	
Trainee console time *	
Anaesthetic time *	
Involvement of an additional surgeon	
Organ injury	
Surgical margin positivity	
Postoperative skin integrity	
C02 pressure used intraoperatively	
Emergency undocking	
Overrunning theatre time	
*Additional KPIs Proposed (Procedure Specific)*	
Length of hospital stay	
Postoperative drain duration	
Patient reported outcomes	
Degree of tilt required for surgery	

Its presents stakeholder views on which key performance indicators (non-procedure-specific) may be appropriate and worthwhile in the oversight of robotic surgical programmes. The table includes a compilation of KPIs proposed by the researchers, and the proportion of respondents in agreement with their inclusion, and also includes additional KPIs proposed by study participants via free-text boxes

The topic of KPIs generated the greatest volume of qualitative data. The need for a carefully thought-out and nuanced approach to their selection and implementation was reiterated by multiple participants, with a number of themes identified, and outlined below.

#### Case complexity

The need for “an acknowledgment/recognition of case difficulty by those not in that particular specialty,” in the instance of recording generic KPIs was stated. In particular, a number of participants stressed that “console time” is incomparable between, for example, “a Whipple’s [procedure] or oesophageal resection,” which may be “routinely > 6 h,” and procedures of a typically shorter duration. Accordingly, it was highlighted that console time may not be an appropriate KPI for these complex major cases, or that it should be appropriately benchmarked.

#### Procedure specificity

A desire for speciality and procedure-specific KPIs was expressed by several participants, who felt that KPIs “should not be a one-size fits all,” but should be “specialty-specific” and could be compared to equivalent “open operations,” for which the example of “[anastomotic] leak rate,” was cited. Several participants also raised the possibility of recording length of stay, postoperative pain, patient-reported and functional outcomes, which would need to be viewed via a procedure-specific lens.

#### Standardisation and validation

The need for individual KPIs to have a “very clear definition,” was highlighted by one respondent, who felt that examples such as “30-day morbidity are too vague.” Another participant emphasised that “reporting standards should be agreed nationally, reported regularly and externally validated intermittently.”

#### Appropriate benchmarking

The need for surgeons to be “carefully benchmarked,” to “include accommodation for early-career learning curves within acceptable parameters and case complexity” was proposed.

#### Danger of KPIs

The risk of the recording of KPIs was stressed by several participants, who cautioned that “prescriptive [generic] KPIs…could be counter-productive.” Concern was expressed regarding the potential for recording of certain KPIs to “influence [intraoperative] decision making” by surgeons, in a way that may not best serve the patient. It was stressed that “focusing too much on time or conversion [to open surgery] is risky…from a safety perspective,” that “some conversions will be for oncology/pathology reasons,” and that creating “avoidance of conversion where same is appropriate,” could be a sequela of declaring open conversion as a KPI. Ultimately, it was voiced that “there is a balance in generating KPIs,” and that a “focus on morbidity” may be more appropriate than “adding excess pressure” with KPIs that could be seen as arbitrary or not universally appropriate or necessarily clinically relevant, such as “to be speedy,” or “to convert to not be over time when…making good progress.”

Furthermore, it was perceived that KPI recording could “[put] people off more complex work, where actually there may be great benefit to robotic approach.”

Ultimately, it was seen as imperative the KPIs do not detract from the “overall aim [of] best possible resection with excellent patient outcomes.”

#### Alternatives to KPIs

One respondent suggested consideration of enhancing focus on continuous surgical learning rather than “summative style” KPIs. For example, it was proposed that rather than a KPI of conversion to an open approach, “encouraging video review with peers is helpful to identify whether there were alternative approaches in a more formative way,” so that appropriate conversion is not discouraged but that the goal is “to elevate everyone’s practice together.”

### Clinical governance structures

**Fig. 2:**
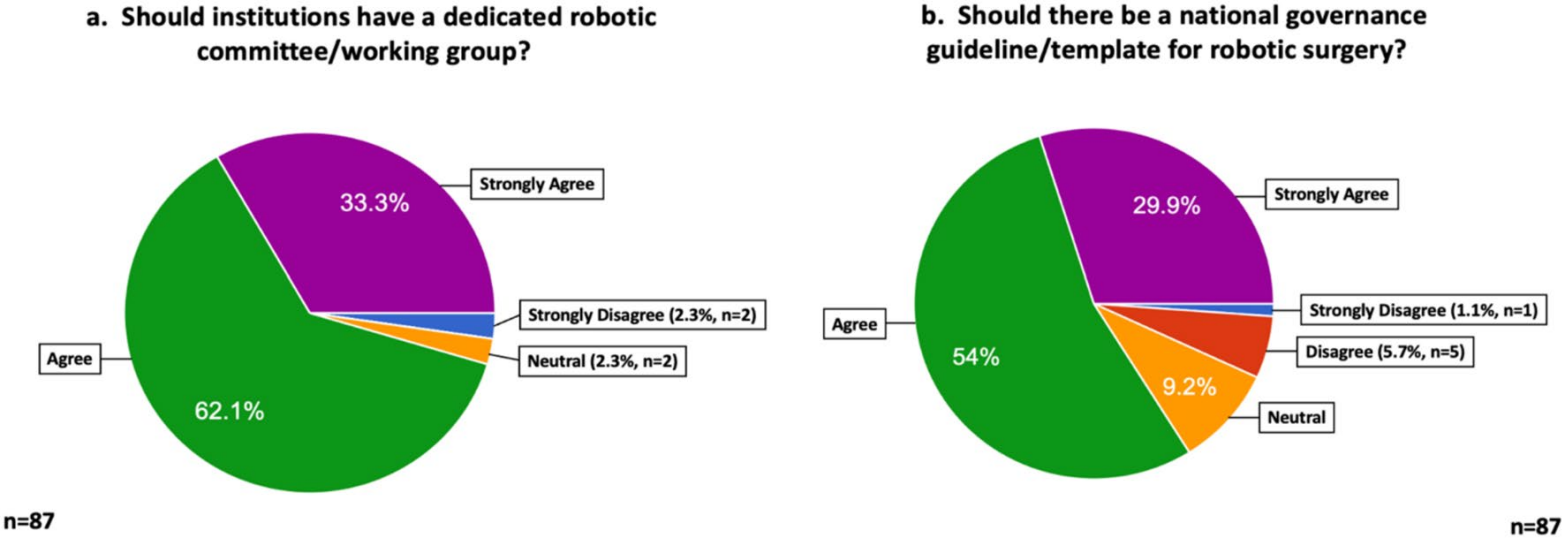
Attitudes towards robotic surgery governance structures and guidelines. It presents the responses and level of agreement on a 5-point Likert scale of respondents towards the existence/establishment of institutional robotic committees to oversee and address robot-specific concepts and issues, and towards the creation of a national guidance document focussed on robotic surgery in Ireland

Respondents largely felt that institutions involved in the delivery of robotic surgery should have a dedicated robotic governance committee/working group that deals with training, mentorship, safety and other robot-specific issues, with 95.4% (*n* = 83) in agreement (Fig. 2). The key personnel on such a committee, as perceived by respondents, are outlined in Supp. Table 1.

A strong support was expressed for the creation of a national guideline or template pertaining to the delivery of robotic surgery in Ireland, with 83.9% (*n* = 73) agreeing that such a document should exist (Fig. 2).

### Additional themes

Thematic analysis was applied to free-text response data. Where relevant, the resultant data has been discussed under the appropriate headings above. A number of additional themes also emerged from this data. These have been summarised in Table 4.

**Table 4:** Additional themes from qualitative data

**Importance of Robot Access** *Respondents highlighted the perceived importance of wide robotic access for both surgeons, to develop and maintain surgical skill, and for patients, to allow choice*
“Equally, for those coming back from robotic fellowships [it’s] important to have access to a robot to progress and maintain skill”
“I’d plan for a significant expansion of robotic practice over the next decade and consider aiming to be one of the first countries where patients get access to new technology based on need rather than availability—several robots in every theatre complex etc.…Ireland is ahead of the curve already and could be a world leader in access”
**Training** *Multiple respondents stressed the need for robotic training, challenges to robotic training and potential approaches to facilitate robotic training*
“[I recommend the] availability of dual consoles. Have ERUS-like proficiency based and simulation based progression/ modules for procedures”
“I think it will be really important for the surgical community to take back control of robotic training in coming years. This has become very industry-driven which can have issues with COI. I think as a community we need to lead out on this rather than leaving credentialing in the hands of industry which may lead to suboptimal safety in some cases. Robotic training is a major challenge for current surgical trainees, who gain very little from bedside assisting, but may struggle to transition to console surgeon in a meaningful way at a pre-fellowship level—particularly with robotic approach being used primarily for major case surgery in Ireland.”
“Just get on with training within the curriculum.”
“Learning [robotics] as a consultant in practice is hugely time constrained [due to] balancing a busy service with this.”
“Trainee involvement and access to console (progression through modular components of various operations such as the approach to RALP for example) could be considered as measurable units/KPIs from a training perspective (a lot of trainees will spend time as bedside assisting and not gaining console time).”
**Business and** **P**u**rchasing** *Respondents commented upon business-related aspects of robot systems, with a view to economic efficiency*
“Choose your robot vendor carefully”
“Is there potential for central purchasing/negotiation on price with manufacturers rather than the weakened negotiation position of an individual hospital”
**FirstAssistant** R**oles** *The potential benefits of dedicated first-assistants, and ways to facilitate development of these roles were frequently cited by respondents*
“We need to create opportunity for Robotic Surgery Nurses to become specialists, ANPs”
“There is a significant requirement for First Assistants in many hospitals. Best to have 1.5 personal permanently contracted. These can be trained from interested OT nursing or other theatre technicians who are already working with and familiar with Robotic surgery. Scope of practice and extended roles would need to be looked at and covered through Competencies and mentoring.”
“[Need to better] consider the role of assistants [when] planning”
“The importance of a well-trained Bedside assistant has yet to be fully recognised in Ireland. Having a constant bedside assistant in the form of an ANP/ SCP has been shown to improve theatre efficiency and safety during robotic surgery. In my opinion it also creates training opportunities for Surgical trainees and the role provides great support for Consultants at the beginning of their Robotic training.”
**Research** *The potential to create a nationwide collaborative research model in robotic surgery, and to broaden the focus of outcome measures was suggested*
“[Have greater focus] on audit and prospective research [in robotic surgery].”
“There needs to be evidenced based data collected re: pain scores and use of analgesia (dose and types) in robotic versus lap/open surgery. This could be one of the strongest patient benefits for robotic assisted surgery, but does not appear to be gathered by surgeons as much as KPI's such as blood loss, conversion rates etc.”
“[I suggest] an all-Ireland collaborative bespoke database involving a series of KPIs which would provide significant evidenced based research and publications.”

## Discussion

Robotic surgery is a rapidly growing field, thus demanding brisk evolution of policies and guidelines. To date, little research has explored the perceptions of key members of the robotic surgical team with regard to robotic surgical practice, oversight and future direction. Using a customised questionnaire, informed by a literature review and expert opinion, and disseminated nationally, we have contributed important preliminary work to this field of research. Strong stakeholder engagement is manifest by the high response rate of robotic surgeons.

Proctoring in robotic surgery may be defined as “observation by another, preferably more experienced, surgeon during initial phase of the [learning curve] of a surgeon learner, to assess knowledge and skills in the use of new equipment or a new technique” [11].

Proctorship arrangements have been advocated with the goal of ensuring adequate robotic skillsets of surgeons and ensuring patient safety during the inexperienced robotic surgeon’s learning curve [8, 11], and some propose are necessary for credentialing [8].

Other studies have reported surgeon satisfaction with proctorship arrangements [12–14], and benefits of more comprehensive mentoring relationships [15]. Our study demonstrates strong support for proctorship and mentorship arrangements in robotic surgery, highlighting the need for institutions and policy makers to safeguard these roles and endeavour to provide for them from a resource perspective.

Since seminal work in the 1970s, both hospital and provider case volume have demonstrated consistent correlation with patient outcomes [16–18], with some authors calling for a “pledge to eliminate low volume surgery” [19]. Unsurprisingly, similar associations between surgical volume and outcome have been confirmed in the setting of robotic surgery [20, 21]. The respondents of our study advocated for regular robotic case performance by robotic surgeons, with 80.3% citing 26 robotic cases per year or a higher figure as the minimum necessary to maintain competence. Further specialty and procedure-specific research may better define necessary case volumes. In addition, the potential role of simulation to maintain proficiency during unavoidable periods of decreased case volume, as used in the aviation industry, merits exploration [3]. Regardless, the surgeons should be encouraged to actively pursue the regular use of robotic systems to maintain proficiency, and hospital management should be aware of this in ensuring regular robot access for all individual robotic surgeons.

The objective performance indicators are recognised as being of great importance in assessing surgeons’ progression through a robotic learning curve, and maintenance of proficiency, and may be viewed as a key facet of quality control within robotic programmes. It is important to ensure such metrics are clinically relevant rather than arbitrary [22]. Whilst our data show a strong majority of stakeholders to support the principle of recording KPIs at both unit-level and surgeon-level, there is some debate with regard to the most appropriate generic KPIs, and also a reminder that a nuanced and considered approach to the topic is necessary to avoid counterproductive instigation of avoidant behaviours amongst surgeons, to patient detriment. Furthermore, development of procedure-specific KPIs, such as the trifecta outcomes already described for certain procedures [23, 24], is desirable in parallel.

A key finding of this study was the strong support voiced by stakeholders for a national guidance document pertaining to the delivery of robotic surgery within Ireland. A number of other nations and specialty societies have already developed such literature [3, 25, 26] with the intention of helping to standardise delivery of robotic surgery, ensure the maintenance of performance standards and to guide policymakers.

There are a number of limitations to our study. As with any questionnaire-based study, there is a risk of respondent bias, although the high response rate amongst robotic surgeons does mitigate against this. In the absence of an established robotic team national communication network, the questionnaire was distributed by senior clinicians via a number of electronic platforms. This methodology may have introduced bias and also means a response cannot be calculated for most groups, as the overall number of professionals who received the questionnaire link is unknown. We acknowledge that this as a limitation. The response rate amongst robotic surgeons is an estimated figure; in the absence of official figures, the denominator was calculated by robotic leads reporting the number of robotic surgeons in their institution/region, all of whom received the survey. Whilst a concerted effort was made to invite a diversity of robotic surgical team members and managers, some groups are underrepresented, and a larger number of responses from nursing colleagues and non-clinical hospital management would be useful. Intentionally, this was a national cross-sectional analysis, and as such all responses are from individuals involved in robotic surgery in Ireland. Whilst many of the surgeon respondents have completed international fellowships, which likely influenced their views on robotic surgery, and whilst a wide range of specialties were captured, further research would be required to confirm generalisability of these findings on an international scale. We attempted to facilitate a broad range of opinions and suggestions, including on subtopics not incorporated in the questionnaire, by means of free-text boxes and qualitative data analysis. Many interesting points were raised. Ideally, further mixed methodology research would be applied to explore these via subsequent semi-structured interviews or focus groups, along with the establishment and input of a Patient and Public Involvement (PPI) panel. This is something the authors plan to consider in the future.

Our work has significant implications for robotic surgery and its evolution in Ireland. It demonstrates a need to take strategic initiatives to foster and encourage mentorship and proctorship relationships for surgeons early on their robotic journey. Similarly, it highlights the need to provide and maintain training opportunities for all members of the robotic surgical team. Whilst further work is required to determine the most appropriate specific KPIs, their applicability to each individual specialty and their oversight, it seems likely that the recording of KPIs will have a role in the future of robotic surgery in Ireland. A strong desire was expressed for a national guidance document on the practice of robotic surgery in Ireland. On this basis, a commitment to produce such a document has been undertaken by the National Leads on Robotic Surgery Group at the Royal College of Surgeons in Ireland.

## Conclusions

This national, pan-specialty robotic surgery stakeholder survey is the first of its kind. It provides important insights into the perceptions of surgeons and key team members regarding the practice and oversight of robotic surgery at both institutional and national levels. A number of important findings have emerged, including high perceived importance of mentorship/proctorship arrangements for early-career robotic surgeons, a belief that an adequate robotic surgical case volume per annum is desirable for surgeons to maintain competence, and that the recording of certain KPIs at both unit-level and surgeon-level is widely supported. Finally, the study has identified a strong majority consensus for institutional governance of robotic surgery and for the development of a national guidelines for the practice of robotic surgery.

## Supplementary Information

Below is the link to the electronic supplementary material.

Supplementary file 1 (DOCX 16 KB)

Supplementary file 2 (PDF 110 KB)

## Data Availability

Raw data is available upon reasonable request.
