# Supplementary material for: A national cross-sectional analysis of stakeholder views regarding the practice and governance of robotic surgery
Source: J Robot Surg. 2025 May 6;19(1):203. doi: 10.1007/s11701-025-02354-w (PMC12055871; doi:10.1007/s11701-025-02354-w)
Supplement: Supplementary file 1 — (DOCX 16 KB) [file 11701_2025_2354_MOESM1_ESM.docx]

**Supp Table 1: Perceived Relevant Members of a Robotic Governance Committee**

| **Professional Role** | **Proportion of Respondents Advocating for Inclusion % (n=)** |
| --- | --- |
| Clinical Director | 72.4% (n=63) |
| Consultant Robotic Surgeons  (all specialties) | 100% (n = 87) |
| Surgical Trainees Rotating through the Unit | 41.4% (n = 36) |
| Assistant Director of Nursing (ADON) | 29.9% (n = 26) |
| Robotic Theatre CNM | 96.6% (n = 84) |
| Central Sterilisation Staff (CSSD) | 47.1% (n = 41) |
| Non-Theatre Based Specialist Nurses Relevant to Robotics (ERAS, Specialty Specific etc) | 42.5% (n = 37) |
| Business Manager | 60.9% (n = 53) |
| Data Manager | 54% (n = 47) |
| Other | Suggested Other Participants  **asterix means suggested by >1 respondent*     - Robotic project lead - Clinical / Safety Engineer* - Anaesthesiologists* - Nursing staff involved in robotic surgery* - ANPs/other first assistants* - Bedflow/bed manager if not covered by other representatives - Post op care unit CNM - Infection control - CEO / representative / business manager / member of Executive management team * - Industry / robotic company representative* - Quality and safety, risk management - Finance* - Business manager and other relevant personnel can be invited in special circumstances e.g. new system or new specialty - HR /Legal representative - Patients / patient advocate / lay representative* - Audiovisual team - Researchers |
