# Supplementary material for: A national cross-sectional analysis of stakeholder views regarding the practice and governance of robotic surgery
Source: J Robot Surg. 2025 May 6;19(1):203. doi: 10.1007/s11701-025-02354-w (PMC12055871; doi:10.1007/s11701-025-02354-w)
Supplement: Supplementary file 2 — (PDF 110 KB) [file 11701_2025_2354_MOESM2_ESM.pdf]

Pan-Specialty Robotic Surgery National Stakeholder Survey 2024

As part of ongoing work by the RCSI National Leads on Robotic Surgery Committee, we aim to explore the perceptions of established robotic surgeons and other relevant stakeholders in Ireland, with regard to optimal governance structures and standard setting in robotic surgery in Ireland.

This survey is aimed at those working in robotic surgery within the Irish public hospital system.

Specifically for surgeon respondents to this survey, your opinion on minimum annual case volume o index procedures, at surgeon- and unit-level, to maintain proficiency within each specialty will also be sought.

Should you have any further queries, please do not hesitate to get in touch.

We would be grateful if you could share your responses as soon as possible, in order to inform emerging work on policy in this area.

Responses are intended to be anonymous; no record will be made of name or email address etc., and data will be analysed in aggregate form. Your consent to processing of your responses will be assumed from completion of the survey.

Thank you very much for your contributions to this important topic.

Kind Regards,

Stefanie Croghan stefaniecroghan@rcsi.ie
Christina Fleming christina.fleming49@gmail.com
Barry McGuire, Chair RCSI National Leads on Robotic Surgery

* Indicates required question

Untitled section

Personal Practice
In this section we will seek demographic and descriptive data regarding your personal surgeon- and unit-level robotic surgery practice.

1. Please select the description that most accurately describes your role *

Mark only one oval.

- Consultant Robotic Surgeon
- Senior Surgical Trainee (ST7/8/Fellow) with robotic console experience
- Senior Surgical Trainee (ST7/8/Fellow) without robotic console experience
- Clinical Nurse Manager in robotic surgery
- ANP/SCP robotic bedside assistant
- Hospital / Group CEO
- Clinical Director
- Industry Partner
- Other: ______________________

2. Which hospital do you work in? *

Mark only one oval.

- St. James's Hospital
- St. Vincent's University Hospital
- The Mater Misericordiae University Hospital
- Tallaght University Hospital
- Beaumont Hospital
- University Hospital Limerick
- Galway University Hospital
- University Hospital Waterford
- Cork University Hospital
- Other: ______________________

3. Have you completed a formal robotic surgery fellowship in your specialty? *

Mark only one oval.

- Yes
- No
- Not applicable

4. Did you receive robotic console training in your surgical training scheme (pre-fellowship)? *

Mark only one oval.

- Yes
- No
- Not applicable

5. For how long have you been practising **surgery** at consultant level? *

Mark only one oval.

- 0 - 5 years
- 6 - 10 years
- 11 - 15 years
- 16 - 20 years
- > 20 years
- Not applicable (not a consultant surgeon)

6. For how long have you been practising **robotic surgery** at consultant level? *

Mark only one oval.

- 0 - 5 years
- 6 - 10 years
- 11 - 15 years
- 16 - 20 years
- > 20 years
- Not applicable (not a consultant surgeon)

7. What is your surgical specialty/sub-specialities? *
(please tick any that apply)

Tick all that apply.

- Not applicable
- General surgery
- Upper GI surgery
- HPB surgery
- Colorectal surgery
- Urology
- Gynaecology
- Thoracic surgery
- Cardiac surgery
- Breast surgery
- Endocrine surgery
- ENT / Head and Neck Surgery
- Other: ______________________

8. What is your personal robotic surgery case volume per year (approximate)? *

Consultants: please respond as cases performed
Trainees, first assistants, PAs and CNMs: please respond as number of cases involved in

Mark only one oval.

- <20
- 21-40
- 41-50
- 51-60
- 61-70
- >70
- Not applicable

9. Question specifically for Robotic (Theatre) CNM:

What is the overall number of robotic surgery cases per year **in your hospital** (approximate)

Mark only one oval.

- <50
- 51-100
- 101-150
- 151-200
- 201-250
- >250
- I don't know
- Not applicable

Surgical Practice in Robotic Surgery

Please state your level of agreement with the following statements.

10. An external proctor (from an outside institution) is a good way to supervise a robotic surgeon in their first number of cases

(Note: An external proctor is an expert in their field picked by the vendor i.e. the robotic surgery company)

Mark only one oval.

- Strongly disagree
- Disagree
- Neutral
- Agree
- Strongly agree
- Unsure

11. An internal proctor (from the same institution) is a good way to supervise a robotic surgeon in their first number of cases

Note: An internal proctor would be a surgeon proficient in robotic surgery, and in the same specialty

Mark only one oval.

- Strongly disagree
- Disagree
- Neutral
- Agree
- Strongly agree
- Unsure

12. Peer to peer mentoring (a more formal relationship where a colleague within the same institution mentors the surgeon early-on in their learning curve until they are signed off as proficient) is a good way to train surgeons beginning robotic surgery or early on their learning curve

Mark only one oval.

- Strongly disagree
- Disagree
- Neutral
- Agree
- Strongly agree
- Unsure

13. In general, what do you think is the approximate **minimum** number of robotic operations a surgeon should perform per year to maintain standards?

Mark only one oval.

- 104 (average 2 per week)
- 52 (average 1 per week)
- 26 (average 1 per fortnight)
- 17 (average 1 every 3 weeks)
- 12 (average 1 per month)
- 6 (average 1 every 2 months)
- Unsure

14. In general, what do you think is the approximate **ideal** number of robotic operations a surgeon should perform per year to maintain standards?

Mark only one oval.

- 104 (average 2 per week)
- 52 (average 1 per week)
- 26 (average 1 per fortnight)
- 17 (average 1 every 3 weeks)
- 12 (average 1 per month)
- 6 (average 1 every 2 months)
- Unsure

Operational Governance

In this section we will explore your opinion on desirable practical operational governance structures for robotic surgery.

Please state your level of agreement with the following statements.

15. Do you think there should be a dedicated robotic surgery operating theatre as opposed to moving the robotic surgery system as needed?

Mark only one oval.

- Yes
- No
- Other: ______________________

16. Procuring a robotic surgery platform is a significant investment for an institution/hospital group. To optimise utilisation, do you agree that the robotic surgery system should be use[illegible] to maximum capacity (i.e. on all working days Mon-Fri)?

Mark only one oval.

- Yes
- No
- Maybe
- Not applicable
- Other: ______________________

17. To maximise utilisation, If you were offered an evening robot slot Mon to Friday would you use it?

Mark only one oval.

- Yes
- No
- Maybe
- Not applicable

18. To maximise utilisation, if you were offered a robot slot on a Saturday would you use it? *

Mark only one oval.

- Yes
- No
- Maybe
- Not applicable

19. In the event of a sudden emergency (e.g. cardiac arrest/unexpected massive bleeding) robotic surgery has increased risk compared to other forms of surgery due to the time it takes to undock/withdraw metal ports/reposition/operating surgeon scrub.

Mark only one oval.

- Strongly disagree
- Disagree
- Neutral
- Agree
- Strongly agree

20. Communication to team members (e.g. bedside assist and circulating nursing staff) can be more challenging during robotic surgery than other forms of surgery.

Mark only one oval.

- Strongly disagree
- Disagree
- Neutral
- Agree
- Strongly agree

21. Emergency undocking should be rehearsed regularly to ensure there is an efficient team response should an emergency occur.

Mark only one oval.

- Strongly disagree
- Disagree
- Neutral
- Agree
- Strongly agree

22. Specific training should be delivered to all members of the operating theatre team regarding effective communication during robotic surgery.

Mark only one oval.

- Strongly disagree
- Disagree
- Neutral
- Agree
- Strongly agree

Clinical Governance

In this section we will explore your opinion on desirable clinical governance structures nationally and locally.

Please state your level of agreement with the following statements.

23. Most hospitals in Ireland have a governance or robotic surgery specific committee but the all differ in their approach.

To guide institutions and align hospitals, there should be some form of a **national governance guideline/templat**e pertaining to the delivery of robotic surgery

Mark only one oval.

- Strongly disagree
- Disagree
- Neutral
- Agree
- Strongly agree

24. Institutions that deliver robotic surgery should have a dedicated robotic governance committee/working group that deal with training/mentoring guidance, safety and any robc specific issues .

Mark only one oval.

- Strongly disagree
- Disagree
- Neutral
- Agree
- Strongly agree

25. Who do you believe should be included in a robotic surgery governance committee? (please select multiple options if appropriate)

Tick all that apply.

- [ ] Clinical Director
- [ ] Consultant robotic surgeons (all specialties)
- [ ] Surgical trainees rotating through the unit
- [ ] Assistant Director of Nursing (ADON)
- [ ] Clinical Nurse Manager (CNM) robotic operating theatre
- [ ] Central sterilisation staff (CSSD)
- [ ] Non-theatre based specialist nurses relevant to robotic surgery e.g. ERAS, specialty specific nursing roles
- [ ] Business manager
- [ ] Data manager
- [ ] Other: ______________________

26. Who would you consider as **other** relevant stakeholders that should be included in a roboti surgery governance committee (if applicable)?

____

____

____

____

____

Audit and Key Performance Indicators

This section will explore your views on regular audit and generic key performance indicators for robotic surgery both at surgeon- and unit-level.

Please state your level of agreement with the following statements

27. Key performance indicators (KPIs) should be recorded at surgeon-level

(Examples of KPIs would include operations of excessive duration, excessive blood loss o transfusion rate, conversions to open, unexpected ICU admission, mortality or readmission).

Mark only one oval.

- Strongly disagree
- Disagree
- Neutral
- Agree
- Strongly agree

28. Key performance indicators (KPIs) should be recorded at unit-level

(Examples of KPIs would include operations of excessive duration, excessive blood loss o transfusion rate, conversions to open, unexpected ICU admission, mortality or readmission).

Mark only one oval.

- Strongly disagree
- Disagree
- Neutral
- Agree
- Strongly agree

29. Please indicate if you agree with inclusion of the following as basic key performance indicators (KPI) within a robotic surgery programme (please select multiple options if appropriate)

Tick all that apply.

- [ ] Any surgery >6 hour duration (console time)
- [ ] Intra-operative transfusion requirement
- [ ] Conversion to open approach
- [ ] Conversion to laparoscopic approach
- [ ] Unexpected return to theatre
- [ ] Unexpected ICU admission
- [ ] 30-day perioperative morbidity
- [ ] 30-day perioperative mortality
- [ ] Other: ______________________

30. Do you have any further comments on the suggested generic KPIs?

____

____

____

____

____

31. Please feel free to share with the research team any further general comments you may have with regard to robotic surgery practice in Ireland.

____

____

____

____

____

Please press submit.

Thank you for taking the time to complete this survey.

Please press submit prior to proceeding.

We will disseminate the findings of this work once it has been fully analysed and we look forward to working with you on continued development of robotic surgery in Ireland.

This content is neither created nor endorsed by Google.

Google Forms
